# Elevated Blood C-Reactive Protein Levels in Patients With Epilepsy: A Systematic Review and Meta-Analysis

**DOI:** 10.3389/fneur.2019.00974

**Published:** 2019-09-18

**Authors:** Rui Zhong, Qingling Chen, Mengmeng Li, Xinyue Zhang, Weihong Lin

**Affiliations:** ^1^Department of Neurology, The First Hospital of Jilin University, Chang Chun, China; ^2^Department of Hepatology, The First Hospital of Jilin University, Chang Chun, China

**Keywords:** C-creative protein, epilepsy, risk, blood levels, meta-analysis

## Abstract

**Background:** In recent years, increasing attention has been paid to the association between C-reactive protein (CRP) levels and epilepsy. However, studies concerning CRP levels in epilepsy have also yielded conflicting results. Thus, the objective of the present study is to systematically review the evidence and conduct a meta-analysis to investigate CRP levels in epileptic patients compared with healthy controls.

**Methods:** A systematic search of PubMed, EMBASE, and the Cochrane Library was performed for eligible studies. Standardized mean differences (SMDs) with 95% confidence intervals (95% CIs) were used as a measure to assess the association between CRP and epilepsy.

**Results:** In total, 16 case–control studies were included in the present meta-analysis, which comprised 1918 individuals. Combined results indicated that epileptic patients had significantly increased CRP levels in peripheral blood compared with healthy controls (SMD = 0.43; 95% CI: 0.19–0.67). In addition, subgroup analyses by age demonstrated that significant differences in blood CRP levels between epileptic patients and healthy controls could be found in adults (SMD = 0.47; 95% CI: 0.21–0.73) but not children (SMD = 0.26; 95% CI: −0.48–0.99).

**Conclusion:** The present meta-analysis shows that the CRP levels in peripheral blood were significantly increased in epileptic patients compared to healthy controls, indicating a significant association between inflammation and epilepsy. Epileptic seizures may be associated with the inflammatory response.

## Highlights

- CRP levels in peripheral blood were significantly increased in epileptic patients compared to healthy controls.
- Significant heterogeneity exists among published studies.
- The elevated levels of C-reactive protein in epileptic patients indicate a significant association between inflammation and epilepsy.

## Introduction

Epilepsy, considered the second most common brain disease, is characterized by the sudden and regular occurrence of seizures (1, 2). It is estimated that more than 65 million individuals are affected by epilepsy worldwide (3). Based on evidence from previous studies, the potential mechanisms for the occurrence and development of epilepsy are wide-ranging and controversial, potentially including oxidative stress, glycation, and methylation capacity (4–8). In recent years, increasing attention has been paid to the association between inflammation and epilepsy; this association is believed to play a key role in the development of epilepsy (9, 10). It has been established that the effect of inflammatory mediators, such as interleukin-6 (IL-6) and tumor necrosis factor α (TNF-α), on the brain is important and harmful. Published studies have reported increased levels of IL-6 and IL-1 in individuals with epilepsy compared with healthy controls, indicating the important role of inflammatory markers in epilepsy (11, 12).

C-reactive protein (CRP), one of the most common biomarkers of inflammation, is widely used to investigate the degree of inflammation (13). CRP has been considered to be associated with the severity of the inflammatory response. High-level evidence from recent meta-analyses indicated that elevated levels of CRP were present in Alzheimer disease (AD) and Parkinson disease (PD) (14–16). Additionally, CRP levels have been identified as a prognostic marker in intracerebral hemorrhage (ICH) by Di Napoli et al. (17). Increased CRP levels during the acute-phase response in ICH may be associated with short- and long-term outcomes such as poststroke epilepsy reflecting the potential association between CRP levels and epilepsy (18). However, studies concerning CRP levels in epilepsy have also yielded conflicting results. A significant association between CRP levels and epilepsy was observed in some studies, while others did not identify consistent results. Thus, the objective of the present study is to systematically review the evidence and conduct a meta-analysis to investigate CRP levels in epileptic patients compared with healthy controls. Additionally, the possible impact of age on CRP levels in epilepsy will be examined by subgroup analysis.

## Methods

The present meta-analysis was performed according to the Preferred Reporting Items for Systematic Reviews and Meta-Analyses guidelines (PRISMA) (19).

### Search Strategy

A systematic review of PubMed, EMBASE, and the Cochrane Library was performed by two reviewers independently. The reference lists of relevant reviews and studies were also screened for eligible studies. The entire time frame from database inception to June 1, 2019, was included. The following search terms were used: “epilepsy” OR “seizure” AND “C-reactive protein” OR “C reactive protein” OR “CRP.” Only studies published in English were included in the present meta-analysis.

### Assessment of Eligibility

In order to be included, a study was required to meet the following inclusion criteria: (1) Design: cross-sectional study, case–control study, or cohort study; (2) Participants: individuals diagnosed with epilepsy or seizure were included in the experimental group, and the control group included those without epilepsy or seizure; (3) Outcome: CRP levels. Duplicate studies were removed first. Reviews, meta-analyses, case reports, letters, and conference abstracts were also excluded. For studies with overlapping patient populations, we included the latest or most complete study.

### Study Selection

The titles and abstracts of all potential studies were screened by two reviewers (Zhong and Chen) working independently. Studies that were obviously irrelevant based on their titles or abstracts were excluded first. Studies that provisionally met the eligibility criteria were assessed for eligibility by examining the full text. Two reviewers (Zhong and Chen) independently checked the articles and resolved disagreements by discussion.

### Data Extraction

The following data were extracted from each included study by two independent reviewers (Zhong and Chen): first author, publication data, country, sample size, outcome, and study design. Specific numerical values of CRP levels were also extracted and used for pooling data. Two investigators independently extracted data from eligible articles, and any discrepant judgments were resolved by discussion.

### Risk of Bias Assessment

The Newcastle-Ottawa Scale (NOS) was applied to assess the quality of the included studies. The NOS score classifies individual studies as having low or high risk of bias across three domains: selection, comparability, and exposure. An included study was defined as high quality when the total score was at least 5 (score of 5–9). Two reviewers independently evaluated the quality of the studies and resolved disagreements by discussion.

### Statistical Analysis

Heterogeneity was assessed by the *P*-value of χ^2^ and the *I*^2^ statistic; the degree of heterogeneity was considered significant if the *I*^2^ statistic was >50% or the *P*-value was >0.5. Data were pooled if a given outcome was reported in at least two studies. The detailed outcome information as reported in the studies was used. Continuous data were described as standardized mean differences (SMDs) with 95% confidence intervals (95% CIs). These values were used to assess the association between CRP levels and epilepsy risk, and the results were presented as forest plots, which included the contribution of each study (weight) to the overall effect. We used random-effects models to pool the results for significant heterogeneity in our meta-analysis; otherwise, a fixed-effects model was applied.

The studies varied in whether their samples were derived from the serum, plasma, or whole blood of epileptic patients; thus, we also conducted a secondary analysis that included only studies whose samples were from serum. Subgroup analyses stratified by age group were further performed.

In addition, a sensitivity analysis was performed by excluding one study at a time to evaluate the stability of the results. Publication bias was assessed by Egger's test if the number of included studies was >10 in the meta-analysis. A funnel plot was also generated. STATA 12 was used for this meta-analysis.

## Results

### Literature Search

**Figure 1:**
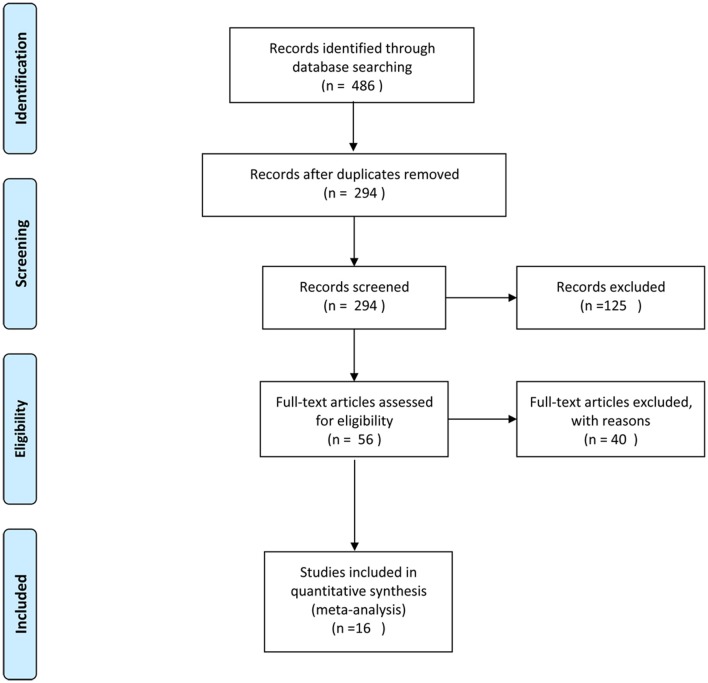
The process of study selection.

The literature search yielded 486 references. A total of 192 duplicate references were removed first. We identified 56 studies as potentially eligible for inclusion after scanning the titles and abstracts. After full-text assessment, we eventually identified 16 studies (20–35) that met the inclusion criteria. A flowchart of the process used to select studies is presented in Figure 1.

### Characteristics and Quality Assessments of Included Studies

In total, 16 case–control studies were included in the present meta-analysis, which comprised 1,918 individuals (1,050 cases and 868 controls) from China (4 studies), Japan (2 studies), Egypt (2 studies), the UK (1 study), the USA (1 study), Italy (1 study), Poland (1 study), South Korea (1 study), Iran (1 study), India (1 study), and Finland (1 study). There was a median sample size of 119 individuals per study. Thirteen studies were conducted in adults, and 3 were conducted in children. Thirteen studies collected their samples from serum, 2 from whole blood, and 1 from plasma. All studies were included in the primary analysis. A secondary analysis was also performed, including only studies whose samples were from serum. The NOS scores of the studies varied from 6 to 8 points (mean score: 6.81), indicating the good quality of the included studies. The basic characteristics of the included studies are provided in Table 1.

**Table 1:** The characteristics of the included studies.

**First author**	**Year**	**Country**	**Age group**	**Number**.		**Outcome**	**Quality**	**Study design**
				**EG**	**CG**			
M. Kopczynska	2018	UK	Adults	157	54	Plasma CRP level	7	Case-control
NA. Meguid	2018	Egypt	Children	30	30	Serum CRP level	7	Case-control
NC Chen	2018	China	Adults	120	40	Blood hs-CRP level	7	Case-control
TC Zhou	2018	China	Adults	58	58	Serum CRP level	6	Case-control
C. Liguori	2017	Italy	Adults	37	20	Serum CRP level	6	Case-control
Kentaro	2017	Japan	Adults	11	8	Serum hs-CRP level	7	Case-control
EP Półtorak	2016	Poland	Adults	21	21	Serum hs-CRP level	6	Case-control
JS Yeom	2016	South Korea	Adults	29	173	Serum CRP level	6	Case-control
A Farhang	2015	Iran	Adults	40	20	Serum CRP level	7	Case-control
N Ishikawa	2014	Japan	Children	29	15	Serum hs-CRP level	7	Case-control
FM. Talaat	2014	Egypt	Adults	40	20	Serum hs-CRP level	7	Case-control
N Sankhyan	2013	India	Children	58	58	Serum hs-CRP level	7	cross-sectional
T Alapirtti	2012	Finland	Adults	31	80	Serum CRP level	8	Case-control
YC Chuang	2011	China	Adults	160	60	Serum hs-CRP level	7	Cross-sectional
S Mintzer	2009	USA	Adults	34	16	Serum CRP level	7	Case-control
TY Tan	2009	China	Adults	195	195	Blood hs-CRP level	7	Case-control

### Results of Meta-Analyses

**Figure 2:**
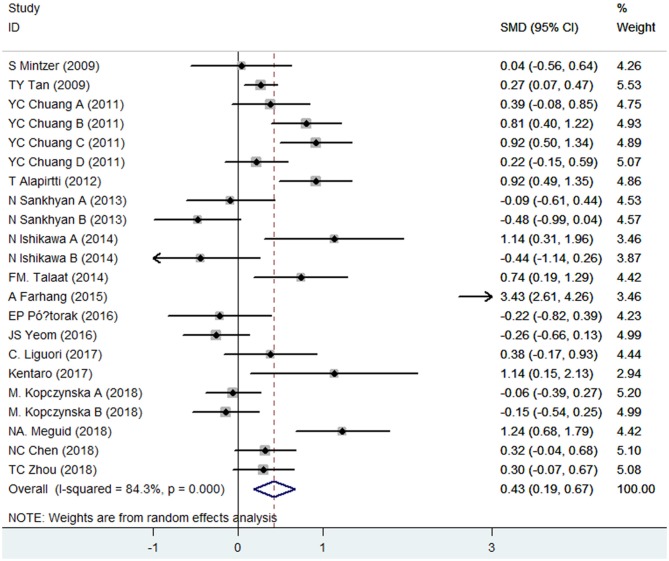
Forest plot of blood C-reactive protein (CRP) levels in epileptic patients and healthy controls. SMD, standardized mean difference; CI, confidence interval.

CRP levels were measured in peripheral blood by a total of 16 case–control studies, whose samples were derived from serum (13 studies), plasma (1 study), or whole blood (2 studies) of epileptic patients. The present meta-analysis involved 1,050 patients and 868 controls. Combined results indicated that epileptic patients had significantly increased CRP levels in peripheral blood compared with healthy controls (SMD = 0.43, 95% CI: 0.19–0.67) (Figure 2). We applied a random-effects model due to the high heterogeneity (*I*^2^ = 84.3%). When removing three studies whose samples were from plasma or whole blood, the secondary analysis demonstrated that CRP levels in serum were also significantly elevated in epileptic patients compared to controls (SMD = 0.53, 95% CI: 0.20–0.85). A random-effects model was also applied due to the high heterogeneity (*I*^2^ = 85.8%).

### Results of Subgroup Analysis Stratified by Age Group

Subgroup analyses by age group were performed as well. Pooled data demonstrated that significant differences in blood CRP levels between epileptic patients and healthy controls were found in adults (SMD = 0.47, 95% CI: 0.21–0.73) rather than children (SMD = 0.26, 95% CI: −0.48–0.99). In other words, adult patients with epilepsy were more likely to have increased levels of CRP than healthy controls. However, this tendency was not observed in children. Random-effect models were used because of the heterogeneity (adults: *I*^2^ = 84.5%; children: *I*^2^ = 84.3%).

### Sensitivity Analysis

**Figure 3:**
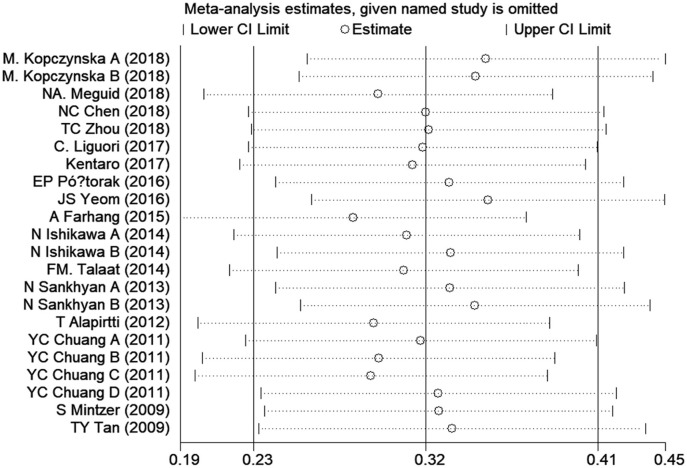
Sensitivity analysis indicates the robustness of the results. CI, confidence interval.

Sensitivity analysis was performed to assess the influence of each individual study on the overall SMD. The stability and reliability of the overall SMD were evaluated by leave-one-out cross-validation, which repeated the analysis after sequential exclusion of each study. The presence of a significant SMD was not changed after serial exclusion of studies (Figure 3). Thus, the results were constant and stable in general.

### Publication Bias

**Figure 4:**
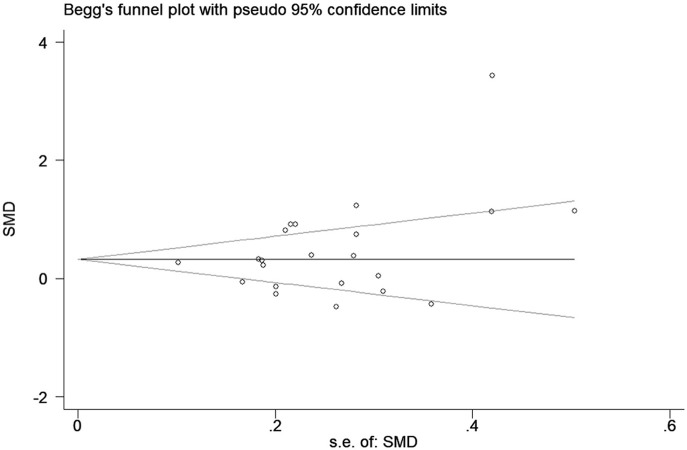
Funnel plot of the selected studies. This suggests publication bias. SMD, standardized mean difference.

A funnel plot for the association between elevated CRP levels and the risk of epilepsy was drawn; visual inspection of this plot did not reveal substantial publication bias (Figure 4). As expected, Egger's test also failed to show publication bias (*p* = 0.22).

## Discussion

In recent years, much attention has been paid to the association between blood CRP levels and the risk of epilepsy. However, this association has not been fully characterized. Our study aimed to systematically review the current evidence and pool the inconsistent data of previous studies focusing on CRP levels in peripheral blood between epileptic patients and controls. The present meta-analysis included 16 studies that recruited 1918 individuals. The findings of the current study mainly showed that epileptic patients had increased levels of CRP in peripheral blood compared with controls. In addition, after removing three studies whose samples were from plasma or whole blood, the secondary analysis demonstrated that CRP levels in serum were also significantly elevated in epileptic patients compared to controls. In addition, subgroup analysis stratified by age group was also conducted, and we found that compared with controls, the CRP levels significantly increased in adult patients but not in child patients. The stability and constancy of the results were demonstrated by sensitivity analysis. Additionally, the funnel plot and Egger's test did not suggest publication bias.

Tan et al. first reported that blood levels of hs-CRP were significantly increased in individuals with epilepsy, which may be associated with the effect of long-term exposure to antiepileptic drugs (AEDs) (35). In line with the findings, Chuang et al. found that patients with epilepsy taking enzyme-inducing AED monotherapy (CBZ and PHT) had more serious elevations of CRP than those with LTG and VPA. Long-term use of AEDs had an important effect on atherosclerosis, which was considered to be associated with inflammatory mechanisms (34). The change in hs-CRP levels may reflect the severity of the inflammatory process. Alapirtti et al. found that CRP levels were significantly increased after index seizure compared with baseline levels (32). Therefore, the CRP levels in epilepsy were also associated with the frequency of seizures. Similar observations were obtained from subsequent (24, 29, 30) studies. However, the opposite results were also reached by some studies that failed to identify a significant association between CRP levels and the risk of epilepsy (26, 31). In addition, Strauss et al. investigated CRP levels in the brain tissue of epileptic and non-epileptic patients and found that decreased levels of CRP were one of the most pronounced epilepsy-associated differences (36). The inconsistent results may be due to the differences in inclusion criteria, age group, and ethnicity. Additionally, because of the limitation of sample size for individual studies, the single study may lack sufficient statistical power to draw a solid conclusion. No high-level evidence on this topic has been obtained from a meta-analysis previously. With the aim of investigating the association between increased CRP levels and the risk of epilepsy, we systematically reviewed the evidence and combined the results of published studies to investigate CRP levels in epileptic patients compared with healthy controls. The significant association between elevated levels of CRP and epilepsy was identified in our meta-analysis.

CRP is considered one of the most important biomarkers of inflammation. In this study, the significant association between increased CRP levels and epilepsy may be explained through the inflammatory mechanism of epilepsy. An increasing body of evidence supports the reciprocal causal link between inflammation and epilepsy (37–39). A recent study also identified the presence of focal and distributed inflammation in epilepsy, as measured by positron emission tomography imaging of translocator protein (40). Acute brain injury such as viral encephalitis and stroke may cause epilepsy whose main driving force is inflammation (39).

Recent evidence supports the idea that acute ischemic and hemorrhagic cerebrovascular diseases are the most common causes of late-onset epilepsy in which the inflammatory response plays an important role (41, 42). It would be significant to explore more inflammatory biomarkers, such as the neutrophil-to-lymphocyte ratio, which may be used to predict the development of epilepsy in acute cerebrovascular diseases and then identify those patients at high risk. Thus, further attention should be paid to finding inflammatory biomarkers that may be used to predict the development of epilepsy in the future. In addition, it was established that epilepsy, especially temporal lobe epilepsy (TLE), was significantly associated with an increased risk of cognitive dysfunction (43). Evidence from previous studies indicated that chronic activation of cytokine-dependent inflammation may lead to cognitive impairment in epilepsy (44). Recent progress has indicated that elevated brain cytokine signaling also causes cognitive impairment (45). CRP, as one of the most common biomarkers of inflammation, may be a potential predictor of subsequent cognitive decline in epilepsy. In recent years, an increasing number of studies have focused on the effect of anti-inflammatory therapy on epilepsy. Recent progress has demonstrated that P2X7 receptor antagonists and monoacylglycerol-lipase (MAGL) are the most effective anti-inflammatory treatments for the acute course of status epilepticus, able to drastically reduce SE duration on a 1-h delay after administration (46–48). Therefore, anti-inflammatory therapies may be an attractive potential choice for epileptic patients with increased CRP levels, although there is a long way to go. In addition, CRP levels can be used to assess the effect of anti-inflammatory treatments. Note that compared with controls, people with epilepsy had an increased risk of subsequent stroke or myocardial infarction (MI), which may be associated with the role of CRP in the development of cardiovascular disease (49, 50). Thus, the increased levels of CRP in epilepsy may predict adverse prognosis, such as a high incidence of stroke or MI.

To our knowledge, the current meta-analysis is the first work focused on the association between increased levels of CRP in peripheral blood and epilepsy. In this work, we combined the results of 16 studies with 1918 individual enrollment, which may enhance the statistical power and the reliability of the findings. The findings showed that significantly increased CRP levels in epilepsy were observed compared to controls. Considering the impact of age on CRP levels in epilepsy, subgroup analysis stratified by age group was performed. A significant association between elevated CRP levels of blood and epilepsy was observed in adult patients but not in child patients. CRP can be used to reflect the degree of inflammation. Thus, the inflammatory response may be more serious in epileptic adults than in epileptic children. The limited number of included studies in the child group was another explanation that may also cause the different results. Thus, the results of subgroup analysis should be interpreted with caution. We also highlight the request to perform more studies to test our results. Generally, the quality of the included studies was high. In addition, the stability and constant of our results were proved by sensitivity analysis. No publication bias was also found by funnel plot and Egger's test.

The present meta-analysis has several limitations. First, a meta-analysis may be biased when the literature search fails to identify all relevant studies. However, access to unpublished articles remains difficult, which may be a potential limitation of our study. Furthermore, only studies published in English were included in our study, which may also have led to bias. Second, the number of included studies based on child patients was limited, and the sample size was small. We highlight the need to conduct more trials to evaluate the association between CRP levels in blood and epilepsy in epileptic children. Third, significant heterogeneity existed among studies. However, we did not identify the source of heterogeneity through a sensitivity analysis and subgroup analysis. The high, significant heterogeneity may be partly explained by the differences in inclusion criteria and basic characteristics across studies. The different detection methods for hs-CRP and CRP among studies may also lead to heterogeneity. Additionally, despite the increased CRP levels observed in epilepsy, CRP levels in epilepsy may be associated with the etiology of epilepsy, the age of epilepsy onset, seizure frequency, and the patient's exposure to AEDs. Subgroup analyses stratified by these potential factors were not performed due to the lack of detailed information. We suggest further studies to assess the effect of the etiology of epilepsy, the age of epilepsy onset, the frequency of seizures, and the exposure to AEDs on CRP levels in epilepsy. CRP is a non-specific biomarker of inflammation for epilepsy that is also elevated in other neurological disorders, such as AD and PD. Increased CRP levels in epilepsy may partly reflect the existence of inflammation. There is a demand for further studies to investigate the association between inflammation and epilepsy. Additionally, the exact role of increased CRP levels in the development of epilepsy remains unclear and requires further research.

## Conclusion

In summary, the present meta-analysis shows that the CRP levels in peripheral blood were significantly increased in epileptic patients compared to healthy controls, indicating a potential association between inflammation and epilepsy. Epileptic seizures may be associated with the inflammatory response. Additionally, the exact role of increased CRP levels in epilepsy remains in question. Further studies are needed to investigate the exact role of CRP in epilepsy.

## Author Contributions

RZ, ML, and XZ: conceptualization. RZ and QC: data curation. RZ: formal analysis and funding acquisition. RZ, QC, and XZ: investigation, methodology, resources, and validation. RZ and ML: project administration. RZ, QC, XZ, ML, XZ, and WL: software. RZ, XZ, and WL: supervision, writing—review, and editing. RZ and XZ: visualization. RZ, QC, and WL: writing—original draft.

### Conflict of Interest Statement

The authors declare that the research was conducted in the absence of any commercial or financial relationships that could be construed as a potential conflict of interest.
